# Upper Gastrointestinal Endoscopic Findings and Their Clinical Correlates in Patients With Liver Cirrhosis in Northern Ghana

**DOI:** 10.7759/cureus.67725

**Published:** 2024-08-25

**Authors:** Bright Oppong, Gordon M Amponsah, Solomon Gyabaah, Mensah K Nicholas, Sarpong Boateng, Prince A Ameyaw, Derrick O Asamoah, Bernard C Nkum

**Affiliations:** 1 Internal Medicine/Gastroenterolgy, Komfo Anokye Teaching Hospital, Kumasi, GHA; 2 Medicine, Kwame Nkrumah University of Science and Technology, Kumasi, GHA; 3 Internal Medicine/Cardiology, Komfo Anokye Teaching Hospital, Kumasi, GHA; 4 Physiology, Kwame Nkrumah University of Science and Technology, Kumasi, GHA; 5 Internal Medicine, Komfo Anokye Teaching Hospital, Kumasi, GHA; 6 Internal Medicine, Bridgeport Hospital/Yale New Haven Health, Bridgeport, USA; 7 Internal Medicine/Cardiology, Kwame Nkrumah University of Science and Technology, Kumasi, GHA; 8 Medicine, Komfo Anokye Teaching Hospital, Kumasi, GHA

**Keywords:** cirrhosis, liver, bleeding, endoscopy, upper gastrointestinal

## Abstract

Background and study aim

Liver cirrhosis causes portal hypertension that leads to dysfunction of the gastrointestinal tract, which may result in complications including upper gastrointestinal (UGI) bleeding. This study sought to determine the prevalence and the clinical correlates of these UGI abnormalities in patients with liver cirrhosis receiving care at the Komfo Anokye Teaching Hospital in Kumasi, Ghana.

Patients and methods

One hundred and forty-five participants with liver cirrhosis were consecutively sampled and clinically evaluated for symptoms and signs of liver cirrhosis and then underwent esophagogastroduodenoscopy (EGD).

Results

The mean age of the respondents was 46.50 ± 12.14 years, with the majority being males (106, 73.10%) and in Child-Pugh class C (111, 76.55%). Fatigue (128, 88.28%) and ascites (127, 87.59%) were the most common symptoms and signs, respectively. Fatigue, itch, and ascites were significantly correlated with the severity of liver cirrhosis, with an adjusted odd ratio (AOR) (confidence interval (CI)) of 3.56 (1.11-11.47), p-value of 0.03, 4.35 (1.34-14.18), p-value of 0.02 and 22.50 (4.88-103.77), p-value < 0.01, respectively. Esophageal varices were the most common UGI endoscopic findings, occurring in 102 (70.34%) patients, and correlated with the severity of liver cirrhosis, AOR (CI) of 5.19 (1.70-15.87), p-value of 0.01. Other common findings included gastritis in 71 (48.97%), portal hypertensive gastropathy in 67 (46.2%), duodenitis in 49 (33.79%), and peptic ulcer in 46 (31.72%).

Conclusions

Fatigue, ascites, and esophageal varices were the most common symptoms, signs, and EGD findings, respectively. Fatigue, itch, ascites, esophageal varices, duodenitis, and gastric antral vascular ectasia correlate with the severity of liver cirrhosis.

## Introduction

Liver cirrhosis is a common endpoint for diseases that lead to chronic inflammation of the liver resulting in fibrosis [1]. Globally, cirrhosis causes 1.16 million deaths, making it the 11th most common cause of death as of 2017 [2]. Liver cirrhosis leads to decreased compliance in the fibrotic liver that gives rise to portal hypertension, which results in complications including ascites, congestive gastroenteropathy, and formation of upper gastrointestinal (UGI) varices, which can lead to UGI bleeding [3]. Massive acute UGI bleeding has poor outcomes, with 38% of fatalities occurring within the first 24 hours [4].

Esophagogastroduodenoscopy (EGD) services are not readily available in most centers in Ghana and sub-Sahara Africa [5,6], making it difficult to perform diagnostic EGD for risk stratification in these patients as directed by guidelines [7], including evaluating and managing emergency UGI bleeding which requires EGD to be done within 24 hours [8]. Consequently, physicians have to manage patients based on clinical findings alone in most cases.

This study sought to describe the prevalence of UGI endoscopic abnormalities and determine the relationship with the severity of liver cirrhosis in patients with liver cirrhosis presenting to the Komfo Anokye Teaching Hospital in Kumasi, Ghana. This information will help guide clinicians in making evidence-based decisions when managing patients with liver cirrhosis, especially in areas where EGD is not readily available.

## Materials and methods

This study was a cross-sectional study involving 145 participants with liver cirrhosis (diagnosed based on clinical, ultrasound, and liver biochemistry findings) who were sampled consecutively at Komfo Anokye Teaching Hospital, the second-largest Hospital in Ghana, which serves as the main referral center for central and northern parts of Ghana with a bed capacity of 1,200. Those who had previously undergone treatment for esophageal varices and those who were on primary prophylaxis for variceal bleeding were excluded from their study.

After informed consent was obtained from participants, information on their socio-demographic characteristics and clinical history was collected using a structured questionnaire. All participants then underwent physical examination where signs of liver cirrhosis such as abdominal swelling/pain, sclera/palmer jaundice, pedal swelling, clubbing, leukonychia, palmar erythema, Dupuytren’s contracture, asterixis, spider naevi, ascites, dry and brittle hair, and hepatic encephalopathy were documented. They again underwent venipuncture for 10 mL of blood, which was used for liver biochemistries and international normalized ratio using Beckman XC700AU biochemistry analyzer (Beckman Coulter, Inc., Brea, CA) and Meril automatic blood coagulometer (Meril Life Sciences, Vapi, India) respectively. Hepatitis B surface antigen and hepatitis C antibody testing were also done.

EGD was performed by an experienced gastroenterologist. Participants were sedated with intravenous midazolam 2.5 mg and hyoscine butylbromide 20 mg for the procedure, and their findings were documented.

Definitions for endoscopic findings

Esophageal varices: prominent submucosal veins that balloon into the lumen of the esophagus.

Gastroesophageal varices: extension of esophageal varices into the stomach.

Duodenitis: the presence of erythema, edema, and friability of the duodenal mucosal surface with or without mucosal hemorrhage or erosions (superficial mucosal breaks).

Ulcer: discontinuation of the inner lining of the gastrointestinal tract that extends into the muscularis mucosa.

Peptic ulcer: ulcer in the stomach (gastric ulcer) and/or an ulcer in the duodenum.

Gastritis: the presence of erythema, edema, and friability of the gastric mucosal surface with or without mucosal hemorrhages or erosions (superficial mucosal breaks).

Gastric antral vascular ectasia (GAVE): dilatation of small blood vessels in the gastric antrum with characteristic “watermelon stripes” appearance.

Isolated gastric varices: gastric varices occur in the absence of extensive esophageal varices.

Lax lower esophageal sphincter: determined by a space around the endoscope on retroflexion at the fundus.

Portal hypertensive gastropathy: friability of the gastric mucosa with ectatic vessels.

Data processing and analysis

Data collected were entered into a database designed with Research Electronic Data Capture (REDCap). The data were exported to STATA (standard edition, version 16.0, StataCorp LLC, College Station, TX) for statistical analysis. Results were enumerated in means and proportions at 95% CI and presented in tables. Both univariate and bivariate analyses were performed. Descriptive analysis was used to summarize the characteristics of respondents. Factors that were significant in bivariate analyses were analyzed using an ordinal regression model to test for the predictors of severe liver disease. A multivariate analysis was performed by using multiple logistic and ordinal regression models. All significant factors were included in the ordinal regression model using a stepwise ordinal logistic method. The unadjusted (crude) and adjusted odds ratios (OR) and 95% confidence intervals (CIs) were reported. The level of significance for association in the bivariate analysis was p < 0.05.

Ethical consideration

Ethical approval for this study was obtained from the Committee of Human Research Publication and Ethics (CHRPE) of Kwame Nkrumah University of Science and Technology and Komfo Anokye Teaching Hospital (CHRPE/AP/045/19). The principles of informed consent and confidentiality were ensured throughout the study.

## Results

A total of 145 participants were included in this study. The mean age of the respondents was 46.5 ± 12.14 years, with a male-to-female distribution of 106 (73.10%) and 39 (26.90%), respectively. The most common etiology of liver cirrhosis was chronic hepatitis B infection occurring in 109 (75.17%) participants, followed by alcohol and hepatitis C, with non-alcoholic fatty liver disease (NAFLD) having the lowest prevalence of 1.38%. Most of the patients at the time of presentation were decompensated, with 111 (76.55%) already in Child-Pugh class C, as shown in Table 1.

**Table 1:** Demographic characteristics, etiology of liver cirrhosis, and clinical state of patients Age - mean age + standard deviation (years); HBsAG - hepatitis B surface antigen; HCVab - hepatitis C virus antibody; NAFLD - non-alcoholic fatty liver disease; SD - standard deviation

Variable	Frequency (n = 145)	Percentage (%)
Age	46.5 ± 12.14	
Gender, male	106	73.10
Education status		
Primary	36	24.83
Junior high school	18	12.41
Senior high school	40	27.59
Tertiary	19	13.10
No formal education	32	22.07
Employment		
Fully employed	44	30.34
Self-employed	94	64.83
Unemployed	2	1.38
Student	2	1.38
Retired	3	2.07
Marital status		
Married	102	70.34
Single	18	12.41
Divorced	12	8.28
Co-habiting	3	2.07
Widow/widower	10	6.90
Etiology of liver cirrhosis		
HbsAg positive	109	75.17
HCVab positive	30	20.69
History of alcohol intake	51	35.15
History of NAFLD	2	1.38
Child-Pugh stage		
Stage A	5	3.45
Stage B	29	20.00
Stage C	111	76.55

The most common presenting symptoms were fatigue, occurring in 128 (88.28%), and fluid congestion, including leg swelling, occurring in 120 (82.76%), and abdominal distention in 117 (80.67%) participants. About half of the participants had a history of blood in stool or vomitus suggestive of UGI bleeding. Fatigue and itch were the presenting symptoms that correlated significantly with the severity of liver cirrhosis with adjusted odd ratio (AOR) (CI) of 3.56 (1.11-11.47), p-value 0.03 and 4.35 (1.34-14.18), and p-value 0.02, respectively, as shown in Table 2.

**Table 2:** Multiple regression model correlating symptoms, signs, and endoscopic findings to the severity of liver cirrhosis (Child-Pugh score) AOR - adjusted odds ratio; GAVE - gastric antral vascular ectasia; LES - low esophageal sphincter; PHG - portal hypertensive gastropathy; UAOR - unadjusted odds ratio; *significant p-value

Variable	Frequency (%) n = 145	UAOR (95% CI)	p-value	AOR (95% CI)	p-value
Symptoms					
Abdominal swelling	117 (80.69)	1.74 (0.70-4.32)	0.230	2.26 (0.73-7.02)	0.157
Blood in stool/vomit	80 (55.17)	1.31 (0.60-2.83)	0.489	1.18 (0.49-2.80)	0.714
Fatigue	128 (88.28)	3.49 (1.23-9.92)	0.019*	3.56 (1.11-11.47)	0.033*
Insomnia	40 (27.59)	0.62 (0.27-1.41)	0.253	0.39 (0.13-1.17)	0.093
Itch/rash	44 (30.34)	3.14 (1.13-8.76)	0.029*	4.35 (1.34-14.18)	0.015*
Jaundice	65 (44.83)	1.68 (0.76-3.71)	0.204	1.51 (0.56-4.10)	0.419
Leg swelling	120 (82.76)	3.31 (1.33-8.24)	0.010*	2.62 (0.95-7.22)	0.063
Dyspepsia	56 (38.62)	0.87 (0.40-1.90)	0.727	0.96 (0.40-2.32)	0.922
Signs					
Clubbing	108 (74.48)	4.55 (1.99-10.42)	<0.001*	3.15 (0.56-17.66)	0.193
Leukonychia	96 (66.21)	2.90 (1.32-6.40)	0.008*	0.93 (0.14-7.18)	0.987
Palmar erythema	42 (28.97)	2.90 (1.04-8.11)	0.042*	0.76 (0.21-2.79)	0.685
Asterixis	17 (11.72)	1.49 (0.40-5.53)	0.550	3.79 (0.62-23.24)	0.150
Spider naevi	29 (20.00)	5.14 (1.16-22.86)	0.032*	2.23 (0.43-1167)	0.341
Dry and brittle hair	93 (64.14)	3.53 (1.59-7.83)	0.002*	1.05 (0.35-5.98)	0.594
Sclera jaundice	54 (37.24)	1.58 (0.69-3.61)	<0.283	1.32 (0.38-4.61)	0.657
Ascites	127 (87.57)	28.42 (7.50-107.68)	<0.001*	22.50 (4.88-103.77)	<0.001*
Esophagogastroduodenoscopy findings					
Esophageal varices	102 (70.34)	3.87 (1.73-8.68)	0.001*	5.19 (1.70-15.87)	0.004*
Gastroesophageal varices	42 (28.97)	3.90 (1.28-11.90)	0.017*	3.10 (0.78-12.33)	0.109
Lax LES tone	36 (24.83)	0.42 (0.18-0.97)	0.042*	0.63 (0.22-1.79)	0.387
PHG	67 (46.21)	1.53 (0.70-3.36)	0.288	0.28 (0.09-0.88)	0.300
GAVE	38 (26.21)	3.31 (1.08-10.14)	0.036*	3.95 (1.08-14.41)	0.037*
Peptic ulcer	46 (31.72)	0.96 (0.42-2.19)	0.928	0.42 (0.15-1.17)	0.097
Gastritis	71 (48.97)	0.81 (0.38-1.75)	0.596	1.30 (0.51-3.32)	0.589
Duodenitis	49 (33.79)	2.35 (0.94-5.87)	0.068	4.73 (1.57-14.27)	0.006*

Ascites was the most frequent sign occurring in 127 (87.57%) of participants, which correlated with the severity of liver cirrhosis AOR (CI) 22.50 (4.88-103.77), p-value < 0.01. Finger clubbing was the second most common sign 108 (74.48%), followed by leukonychia and jaundice. However, there was no significant association between these signs and the severity of liver cirrhosis, as shown in Table 2.

## Discussion

In this study, we found the mean age of developing liver cirrhosis to be 46.50 ± 12.14 years, with more males being affected than females. Chronic hepatitis B infection was the most common cause of liver cirrhosis, and most of the participants reported late to the hospital already in a decompensated condition in Child-Pugh class C. Fatigue, itch/rash, ascites, esophageal varices, GAVE, and duodenitis were the factors that were associated with the severity of liver cirrhosis. The age and gender distribution of participants in this study, of which the majority were predominantly young men, mirrors what has been found in southern Ghana [9] and also other countries in the sub-Saharan subregion, including Nigeria [10], Uganda [11], and Côte d'Ivoire [12]. This is of economic importance as relatively young men who belong to the productive group are affected more in the subregion compared to the West, where liver cirrhosis occurs a decade later [13]. This may be explained by the fact that the most common cause of cirrhosis in Ghana, as seen in this study and similar studies in Ghana [9] and the subregion [14], is chronic hepatitis B infection, which tends to be a result of maternal-to-child transmission in Africa [15] thus occurring early and causing liver damage earlier than those in the West where the likely liver cirrhosis etiology is alcohol, hepatitis C, and NAFLD all of which tend occur later in life [16].

Fatigue was the most common symptom found in 88.28% of participants. Fatigue is a common symptom in chronic conditions, including liver cirrhosis. The liver regulates energy metabolism, and disturbances in liver function impair this critical function, which can lead to reduced energy levels and fatigue [17]. Certain inflammatory markers, including tumor necrosis factor and interleukins, which become elevated in liver cirrhosis, also play roles in stimulating certain areas of the brain, which can lead to fatigue [18,19]. This study found that fatigue correlates with the severity of liver cirrhosis. A similar finding was observed by Lui et al., who found this relationship in young people with liver cirrhosis [20]. Fluid overload (evidenced by pedal edema and abdominal distension as presenting complaints and ascites on physical examination) was a common symptom/sign seen in this study. This was the case because most of the patients who were enrolled in this study had decompensated and, as such, were likely to have evidence of fluid retention, highlighting a defect in the health-seeking behavior of patients in sub-Sahara Africa with the tendency to present to the hospital when the disease is advanced [21]. This poor health-seeking behavior is influenced by socioeconomic challenges in the subregion [22].

Esophageal varices were the most predominant EGD finding in this study, with a prevalence of 70.34%. This high prevalence of esophageal varices in patients with cirrhosis at our tertiary hospital had a similar trajectory as what was found in southern Ghana, where it was as high as 90.6% [23]. This is also similar to findings from other neighboring countries in West Africa, such as Nigeria and Côte d'Ivoire, where their hospital prevalence of esophageal varices in patients with cirrhosis was 75% and 76.6%, respectively [12,24]. This high prevalence of esophageal varices can be explained by the fact that most of the participants in the study and also those in the aforementioned countries were decompensated at presentation, with as much as 76.5% already in Child-Pugh class C in our study. The risk of developing and progression of esophageal varices is directly related to the stage of liver cirrhosis [25].

Other important UGI endoscopic findings that were found in this study included gastritis, duodenitis, peptic ulcer, and gastric antral ectasia. These findings are important because they may be the source of bleeding (non-variceal) in patients with liver cirrhosis. Patients with cirrhosis have a higher risk of developing peptic ulcers compared to the general population [26]. Gado et al. in Egypt, for instance, found that patients with cirrhosis who develop peptic ulcer disease are at an increased risk of bleeding than those in non-cirrhotic patients, and 60% of non-variceal bleeding in patients with cirrhosis was due to peptic ulcer [27].

The presence of peptic ulcers in cirrhotic patients may be associated with some ulcerogenic factors that are specific to patients with liver cirrhosis. Among the proposed factors are hypergastrinemia, decreased gastric prostaglandin E2 levels, and the observed portosystemic shunting in liver cirrhosis, which may prevent ulcerogenic factors from being cleared by the liver [28,29]. Also, it has been observed that the presence of portal hypertension and PHG may predispose the gastric mucosa and duodenal mucosa to damage by ulcerogenic factors and impair the ability to repair the damage [30]. In this present study, portal hypertensive gastropathy was observed in 46.21% of our patients, and this may explain the high prevalence of gastroduodenal ulcers observed. Given the high prevalence of risk of UGI bleeding from both variceal and non-variceal sources in this study, it was not surprising that about half of the patients had a history of gastrointestinal bleeding.

Limitations

The use of an antispasmodic, hyoscine butyl bromide during the endoscopic procedure could have affected the laxity of the lower esophageal sphincter. Also, this study is hospital-based, and the findings may not be generalizable to the community.

## Conclusions

There is a high prevalence of UGI abnormalities in patients with liver cirrhosis, including esophageal varices and non-variceal endoscopic findings, which may increase the risk of gastrointestinal bleeding. Fatigue, ascites, and esophageal varices were the most common symptoms, signs, and EGD findings, respectively. Some signs and symptoms of liver cirrhosis, including fatigue, itch, ascites, esophageal varices, duodenitis, and GAVE, correlate with the severity of liver cirrhosis. Physicians can take into consideration these correlations when managing patients with liver cirrhosis.
